# Supplementary material for: Damped circadian oscillation in the absence of KaiA in Synechococcus
Source: Nat Commun. 2020 May 7;11:2242. doi: 10.1038/s41467-020-16087-x (PMC7205874; doi:10.1038/s41467-020-16087-x)
Supplement: Supplementary file 1 — Supplementary Information [file 41467_2020_16087_MOESM1_ESM.pdf]

Damped circadian oscillation in the absence of KaiA in *Synechococcus*

Kawamoto *et al*.

Supplementary Information

Supplementary Information

**Strain name**	**Genotype**	**Host strain**	**Plasmid**	**References**
ILC976 (NUC 42)	P_{kaiBC}::*luxA*, Cm^R	-	-	1
ILC128 (NUC 43)	Δ*kaiABC*;P_{kaiBC}::*luxAB*, Km^R, Cm^R	ILC976	pDkaiABC	1
ILC1018	Δ*kaiABC*;P_{kaiBC}::*luxAB*, Sp^R, Cm^R	ILC976	pIL764	This work
ILC653	Δ*kaiBC*;P_{kaiBC}::*luxAB*, Sp^R, Cm^R	ILC976	No name	3
ILC568	*kaiA*$^-$;P_{kaiBC}::*luxAB*, Sp^R, Cm^R	ILC128	pIL1047	2
ILC770	*kaiA*$^-$(double nonsense) ;P_{kaiBC}::*luxAB*, Sp^R, Cm^R	ILC128	pIL813	This work
ILC767	*kaiA*$^-$; *kaiB*$^-$;P_{kaiBC}::*luxAB*, Sp^R, Cm^R	ILC128	pIL810	This work
ILC662	*kaiA*$^-$; Δ*kaiC*;P_{kaiBC}::*luxAB*, Km^R, Cm^R	ILC568	pDkaiC	2
ILC661	*kaiA*$^-$; Δ*sasA*;P_{kaiBC}::*luxAB*, Sp^R, Km^R, Cm^R	ILC568	pDsaskm	4
ILC680	*kaiA*$^-$; *cikA*$^-$;P_{kaiBC}::*luxAB*, Sp^R, Gm^R, Cm^R	ILC568	pAM2152	5
ILC1039	*kaiA*$^-$; *labA*$^-$;P_{kaiBC}::*luxAB*, Sp^R, Km^R, Cm^R	ILC568	pDlabA(Km)	6
ILC1468	Δ*kaiBC*;P_{trc}::*kaiBC*;P_{kaiBC}::*luxAB*, Sp^R, Km^R, Cm^R	ILC653	pNS2Ptrc-kaiBC	7
ILC1069	Δ*kaiA*;P_{trc}::*kaiBC*;P_{kaiBC}::*luxAB*, Sp^R, Km^R, Cm^R	ILC1018	pNS2Ptrc-kaiBC	7
ILC1465	Δ*kaiA*;P_{0050}::*kaiBC*;P_{kaiBC}::*luxAB*, Sp^R, Em^R, Cm^R	ILC1018	pIL958	This work
ILC1019	Δ*kaiA*;D4::*kaiBC*;P_{kaiBC}::*luxAB*, Sp^R, Km^R, Cm^R	ILC1018	No name	8
ILC541	*kaiA*$^-$; *kaiC*^{EE};P_{kaiBC}::*luxAB*, Sp^R, Km^R	ILC128	pIL698	This work
ILC785	*kaiA*$^-$; *kaiC*^{AA};P_{kaiBC}::*luxAB*, Sp^R, Km^R	ILC128	pIL851	This work
ILC778	*kaiA*$^-$; *kaiC*^{A87V};P_{kaiBC}::*luxAB*, Sp^R, Km^R	ILC128	pIL840	This work
ILC766	*kaiA*$^-$; *kaiC*^{S157P};P_{kaiBC}::*luxAB*, Sp^R, Km^R	ILC128	pIL777	This work
ILC776	*kaiA*$^-$; *kaiC*^{R321Q};P_{kaiBC}::*luxAB*, Sp^R, Km^R	ILC128	pIL838	This work
ILC765	*kaiA*$^-$; *kaiC*^{R393C};P_{kaiBC}::*luxAB*, Sp^R, Km^R	ILC128	pIL776	This work
ILC777	*kaiA*$^-$; *kaiC*^{F470Y};P_{kaiBC}::*luxAB*, Sp^R, Km^R	ILC128	pIL839	This work

Supplementary Table 1. Strains used in this study.

Genotype	Fitted period (h)	Autocorrelation (h)	Peak-to-peak (h)	Damping rate × 10^{-2}(1/h)
WT (25°C)	25.1±0.26	25.0±0.33	25.9±1.50	0.154±0.097
WT (27°C)	25.3±0.10	25.3±0.00	25.1±0.48	0.031±0.018
WT (30°C)	25.1±0.18	25.1±0.19	25.1±0.42	0.052±0.030
WT (32°C)	25.2±0.04	25.3±0.00	25.5±0.32	0.093±0.023
$kaiC^{A87V}$	22.4	22.3	22.0	0.055
$kaiC^{S157P}$	22.3	22.3	21.0	0.0
$kaiC^{R321Q}$	23.8	23.7	21.0	1.186
$kaiC^{R393C}$	15.1	15.0	15.0	0.106
$kaiC^{F470Y}$	16.7	16.7	17.0	0.005
$kaiC^{EE}$	43.8±0.72	44.0±1.41	40±1.89	1.583±0.880
$kaiA^{-}$ (25°C)	27.3±1.24	27.7±1.69	30.2±1.44	7.150±0.820
$kaiA^{-}$ (27°C)	27.3±0.74	26.3±0.67	27.0±1.03	4.482±0.341
$kaiA^{-}$ (30°C)	25.8±1.31	24.8±1.29	24.0±1.51	5.732±0.844
$kaiA^{-}$ (32°C)	26.9±0.88	25.1±0.83	25.0±0.66	7.787±0.386
$kaiA^{-};kaiC^{A87V}$	25.1±0.63	24.6±0.51	25.2±0.16	4.558±0.275
$kaiA^{-};kaiC^{S157P}$	22.1±0.57	20.7±0.58	21.4±0.34	8.346±0.371
$kaiA^{-};kaiC^{R321Q}$	30.6±1.01	29.4±0.69	28.1±1.96	2.749±0.632
$kaiA^{-};kaiC^{R393C}$	25.5±0.70	24.6±0.69	24.8±0.57	5.470±0.125
$kaiA^{-};kaiC^{F470Y}$	26.3±1.08	25.4±0.69	25.7±0.269	6.865±0.496
$kaiA^{-};kaiC^{EE}$	24.7±0.60	21.9±0.38	21.2±1.112	9.052±0.360

Supplementary Table 2. Periods estimated by three methods and damping rates.

Supplementary Figure 1

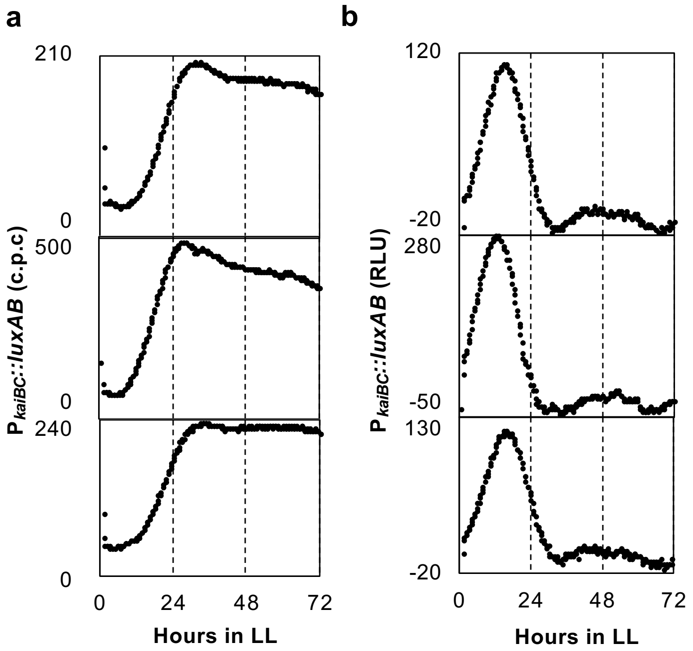

Supplementary Figure 1. (**a**) Three replicated bioluminescence profiles in the *kaiA*⁻ strain under continuous low light conditions (~15 μmol photon·m^{-2}·s^{-1}) after entrainment to two light-dark cycles. (**b**) Detrended profiles of (**a**). Because the second peaks were not observed reproducibly, period lengths were not calculated.

Supplementary Figure 2

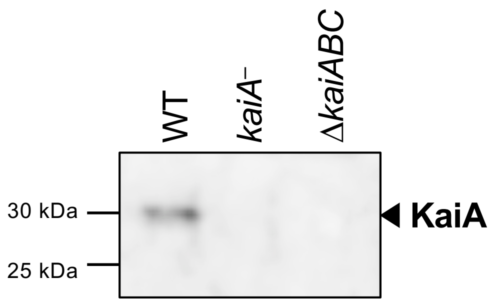

Supplementary Figure 2. Western blotting analysis using anti-KaiA antiserum for the wild-type (WT), *kaiA*$^{-}$ and Δ*kaiABC* strains used in the present study. One of two independent studies is shown. In both experiments, signal for KaiA was exclusively detected in WT.

Supplementary Figure 3

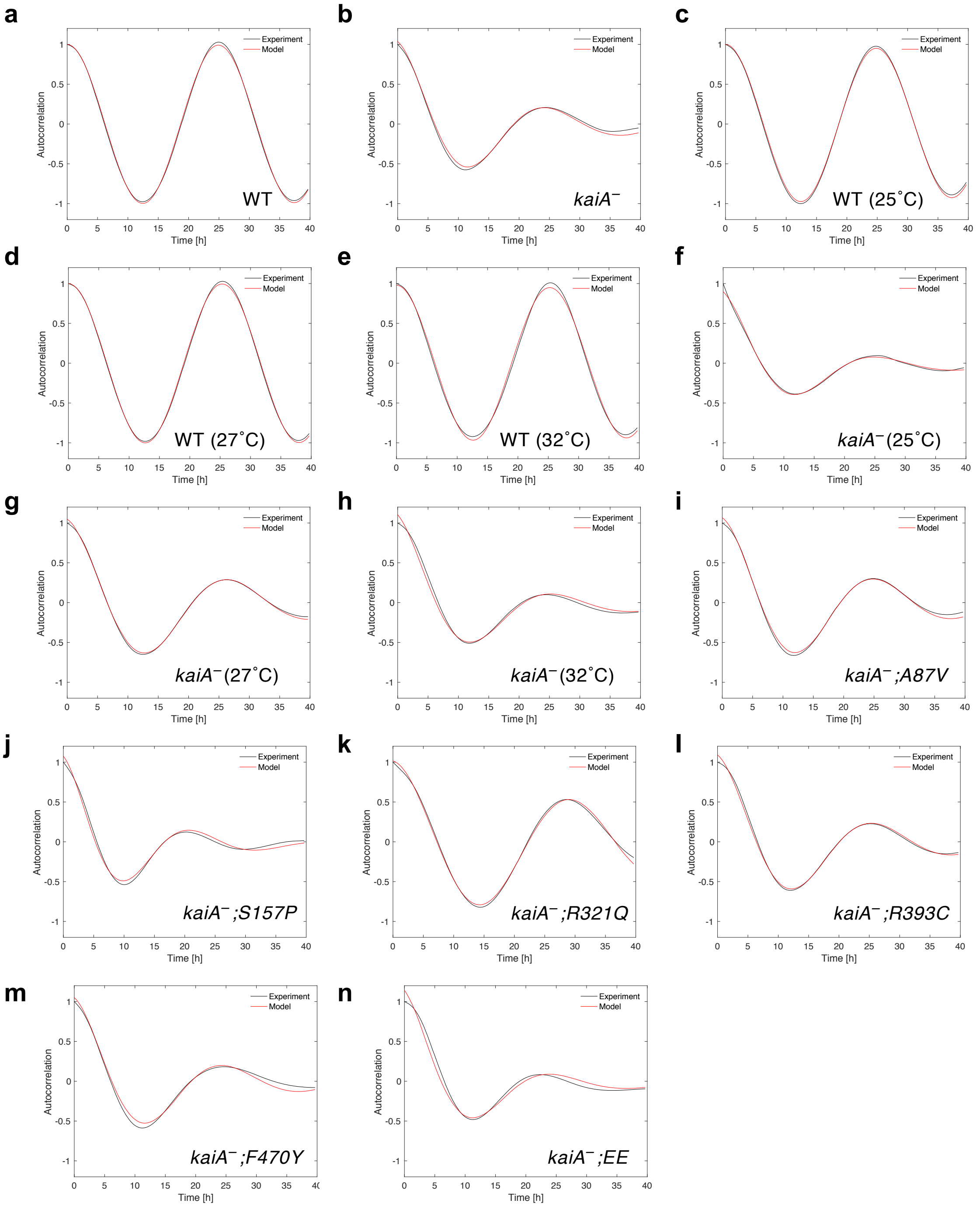

Supplementary Figure 3. Fitting the auto-correlation function for experimental data. (**a**) WT at 30° C (standard condition), (**b**) $kaiA^{-}$ at C (standard condition), (**c**) WT at 25° C, (**d**) WT at 27° C, (**e**) WT at 32° C, (**f**) $kaiA^{-}$ at 25° C, (**g**) $kaiA^{-}$ at 27° C, (**h**) $kaiA^{-}$ at 32° C, (**i**) $kaiA^{-}$ *;A87V* at 30° C, (**j**) $kaiA^{-};kaiC^{S157P}$ at 30° C, (**k**) $kaiA^{-};kaiC^{R321Q}$ at 30° C, (**l**) $kaiA^{-};kaiC^{R393C}$ at 30° C, (**m**) $kaiA^{-};kaiC^{F470Y}$ at 30° C, and (**n**) $kaiA^{-};kaiC^{EE}$ at 30° C.

Supplementary Figure 4

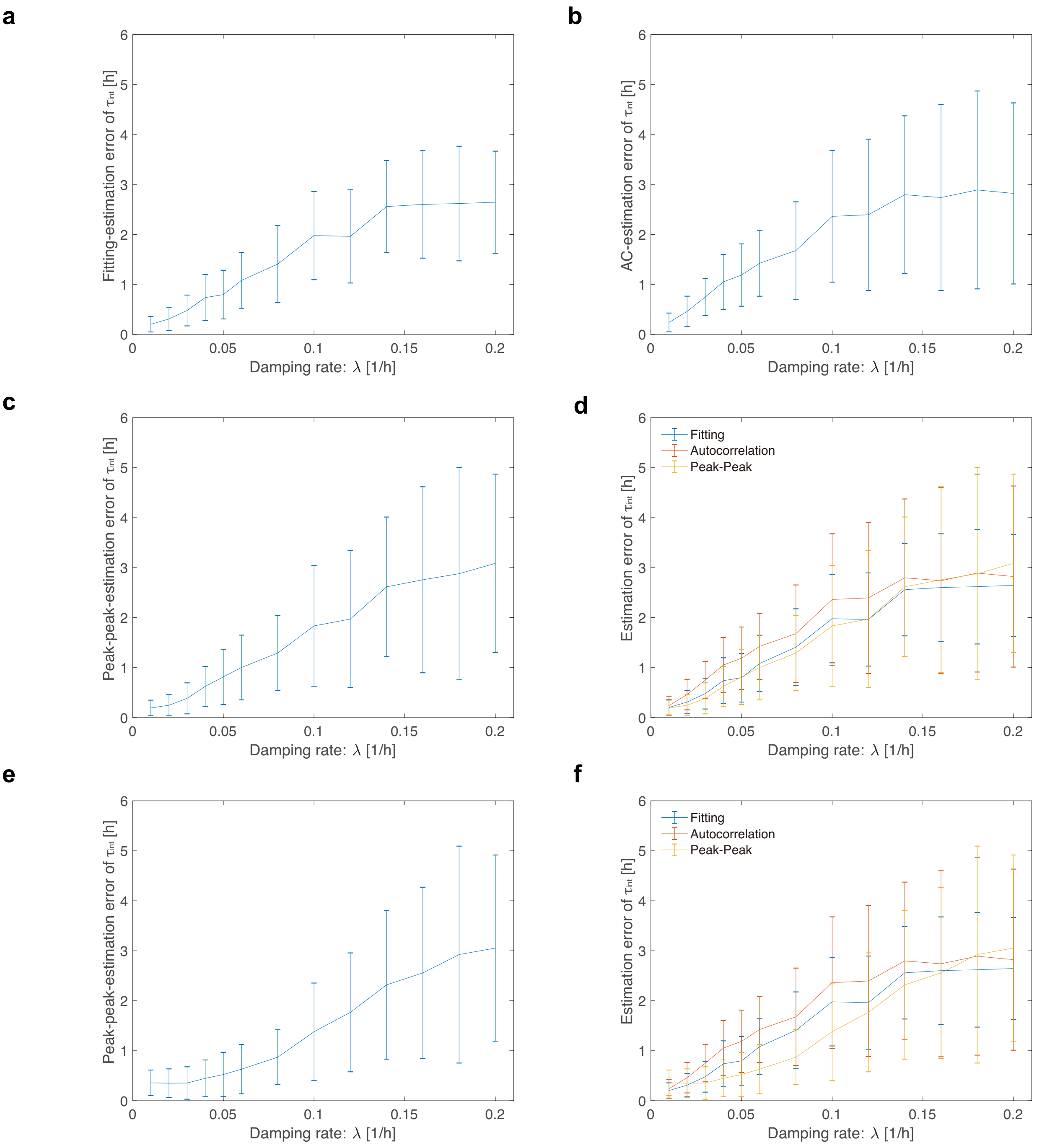

Supplementary Figure 4. Dependence of the estimation error of period t_{int} on the damping rate l (average and standard deviation of 100 trials). (**a**) Estimated by the method of Westermark *et al*. (supporting reference 5). (**b**) Estimated by the peak of the autocorrelation function. (**c**) Estimated by the initial two peak-to-peak intervals (average of the first and second peak-to-peak intervals). (**d**) Comparison of (**a**), (**b**) and (**c**). (**e**) Estimated by the first peak-to-peak intervals. (**f**) Comparison of (**a**), (**b**) and (**e**).

Supplementary Figure 5

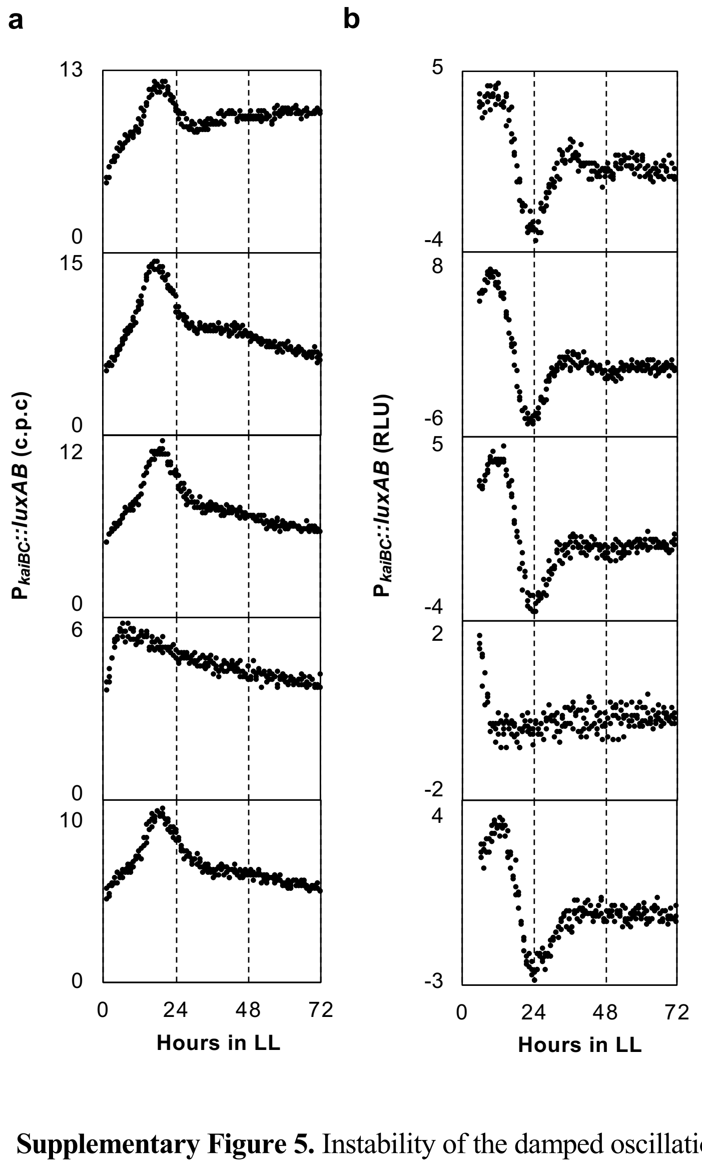

Supplementary Figure 5. Instability of the damped oscillation of *kaiA*⁻ at 35° C. (**a**) Five replicated bioluminescence profiles in the *kaiA*⁻ strain under continuous light at 35° C. (**b**) Detrended profiles of (**a**). Because the second peaks were not observed reproducibly, period lengths were not calculated.

Supplementary Figure 6

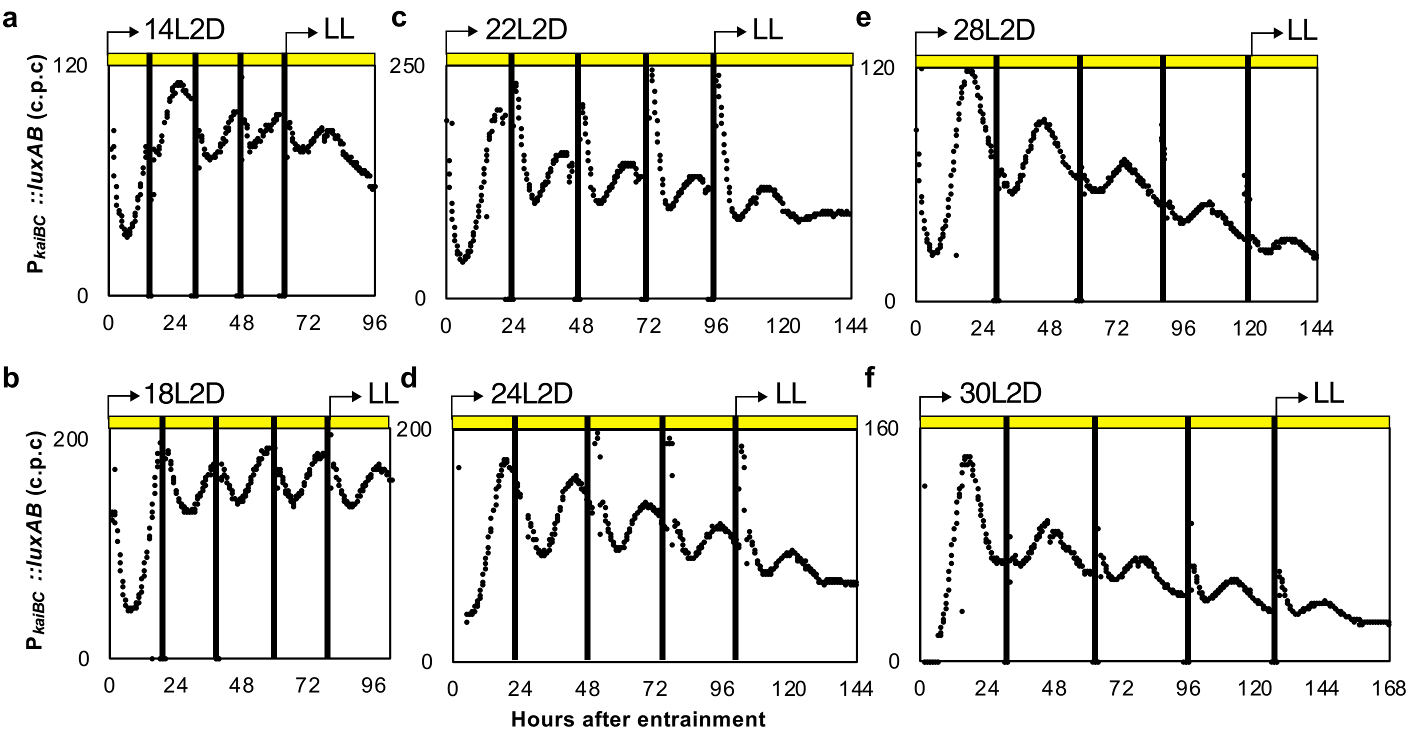

Supplementary Figure 6. Bioluminescence profiles in the *kaiA*⁻ strain were resonated with 2-h dark pulses with a period ranging from 16 to 32 h. After two 12-h:12-h light–dark (LD) cycles, bioluminescence was monitored while 2-h dark pulses were given four times repeatedly over a period of 16 (**a**), 20 (**b**), 24 (**c**), 26 (**d**), 30 (**e**) or 32 h (**f**). Black and bars indicate the durations of dark and light exposure, respectively.

Supplementary Figure 7

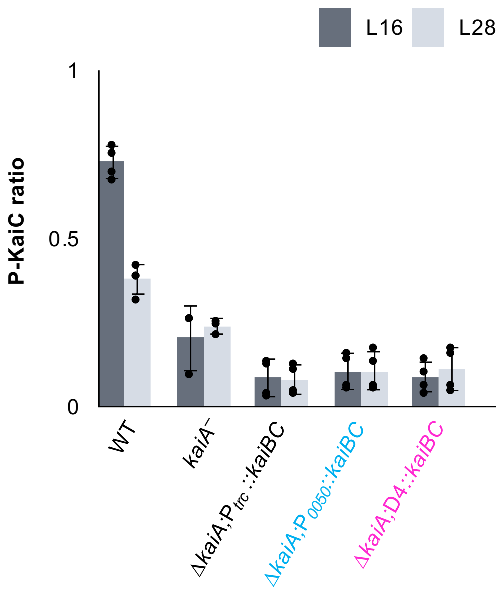

Supplementary Figure 7. The P-KaiC/total KaiC ratio was quantified via densitometric analysis of Western blotting data in the wild-type (WT) and *kaiA*-null strains (*n* = 4 or 3, representative western data are shown in Fig. 4g). Black and gray bars represent the mean values at hour 16 and 28 in the light, respectively. Dot plots represent density of individual bands, and error bars indicate s.d.

Supplementary Figure 8

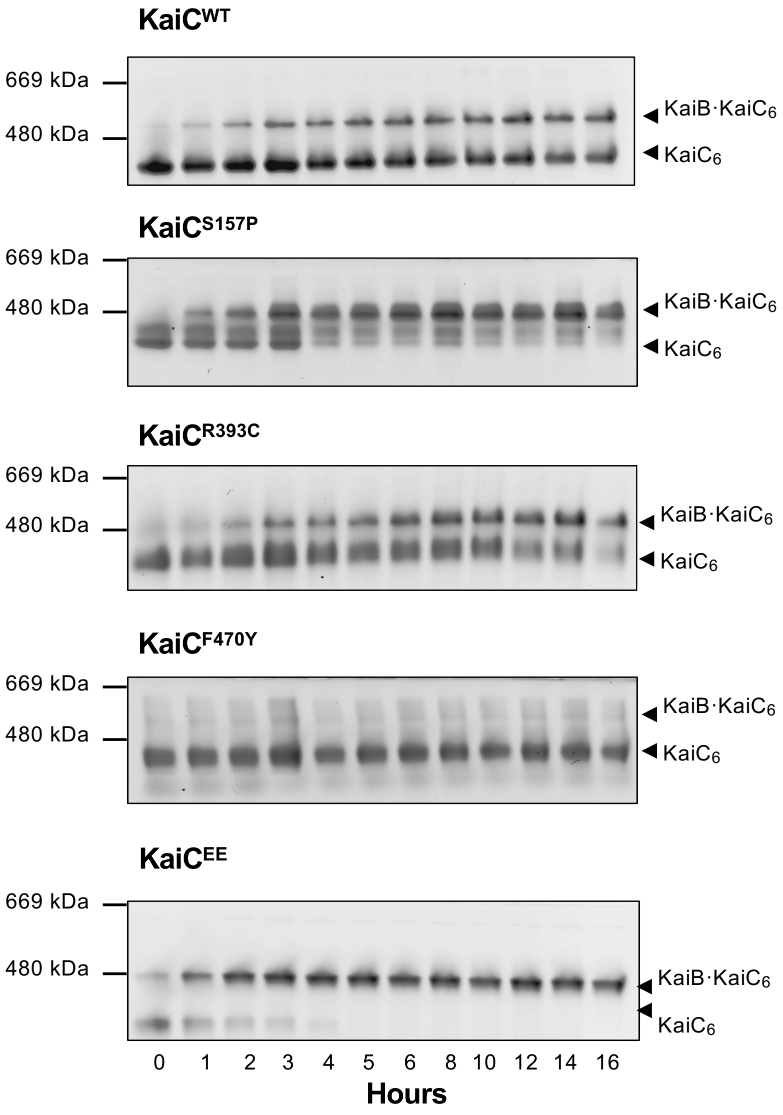

Supplementary Figure 8. Dynamics of KaiB-KaiC complex formation as analyzed via Native PAGE. Recombinant KaiB and the wild-type (WT) or mutant KaiC proteins were mixed at hour 0 and incubated at 30° C ($n = 2$). For details, see Materials and Methods. Upper and lower bands represent KaiB-KaiC and KaiC complexes, respectively. $KaiC_6$ refers to the KaiC hexamer.

Supplementary Figure 9

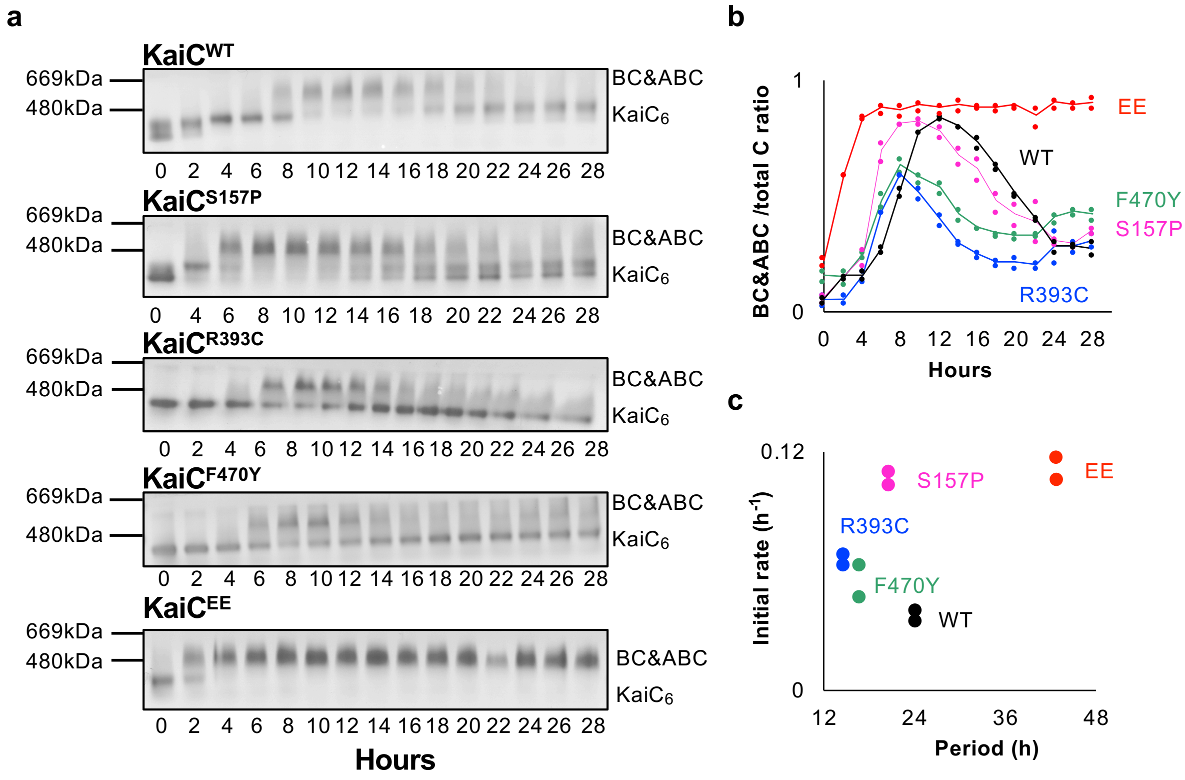

Supplementary Figure 9. KaiA-KaiB-KaiC complex formation analysed via Native PAGE. (**a**) Dynamics of KaiA-KaiB-KaiC complex formation as analyzed via Native PAGE. Recombinant KaiA, KaiB and the wild-type (WT) or mutant KaiC proteins were mixed at hour 0 and incubated at 30° C. The upper bands contain both KaiBC and KaiABC complexes, whereas the lower bands represent the KaiC hexamer. Representative data of duplicated experiments are shown. (**b**) Densitometric analysis of the Native PAGE gels ($n = 2$). Dot plots indicate each experimental value, and the curved lines indicate mean values. (**c**) The calculated KaiB-KaiC or KaiA-KaiB-KaiC complex formation rates between hour 0 and 6 are plotted against the period lengths of the intact (KaiABC-containing) sustained oscillator with each indicated *kaiC* mutation.

Supplementary Figure 10

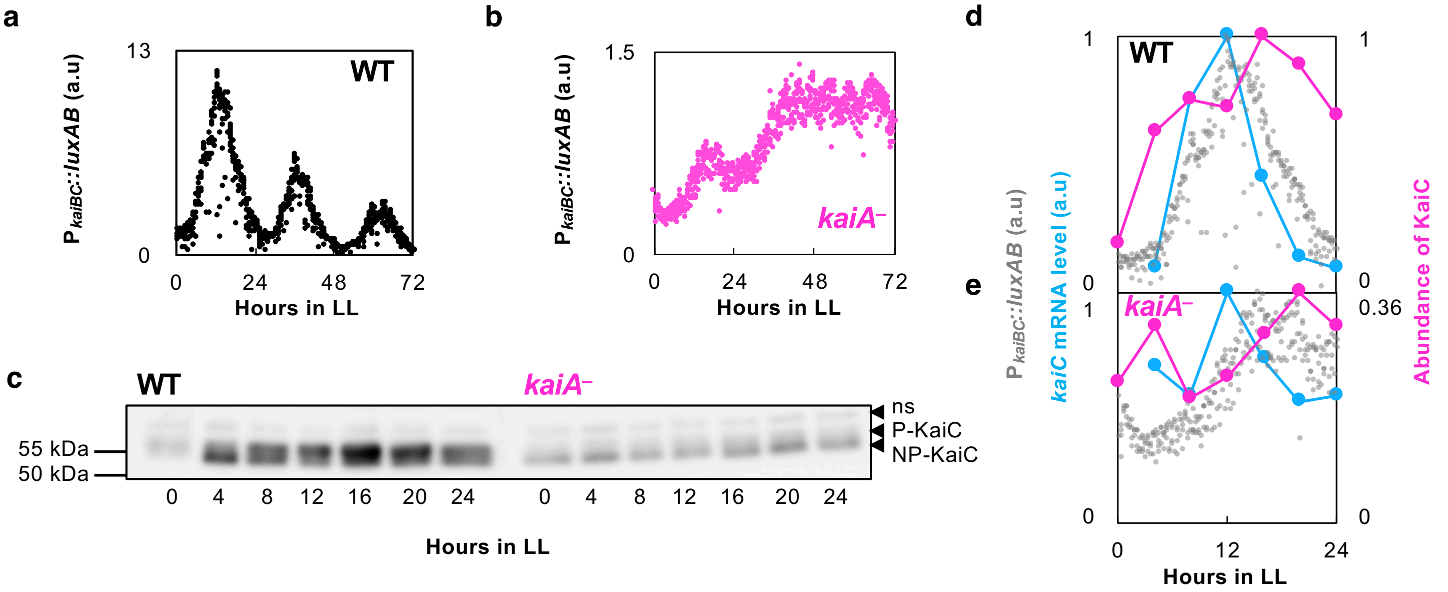

Supplementary Figure 10. Profiles of bioluminescences, *kaiBC* mRNAs and KaiC in WT and *kaiA*⁻ in LL. (a) Bioluminescence profiles of the wild type and (**b**) *kaiA*⁻ strains in continuous liquid culture used for sampling. (**c**) Western profiles for KaiC. (**d**, **e**) Combined data of bioluminescence (promoter activity, gray), *kaiBC* mRNA by qPCR (cyan), and KaiC protein level (magenta) in WT (**d**) and (**e**) *kaiA*⁻ ($n = 1$).

Supplementary References

1. Nishimura, H., et al., Mutations in KaiA, a clock protein, extend the period of circadian rhythm in the cyanobacterium *Synechococcus elongatus* PCC 7942. *Microbiology* **148**, 2903-2909 (2002).
2. Ishiura, M., *et al.*, Expression of a gene cluster *kaiABC* as a circadian feedback process in cyanobacteria. *Science* **281**, 1519-1523 (1998).
3. Nakahira, Y., *et al*., Global gene repression by KaiC as a master process of prokaryotic circadian system. *Proc Natl Acad Sci U S A* **101**, 881-885 (2004).
4. Iwasaki, H., *et al*., A KaiC-interacting sensory histidine kinase, SasA, necessary to sustain robust circadian oscillation in cyanobacteria. *Cell* **101**, 223-233 (2000).
5. Schmitz, O., Katayama, M., Williams, S. B., Kondo, T., Golden, S. S. CikA, a bacteriophytochrome that resets the cyanobacterial circadian clock. *Science* **289**, 765-768 (2000).
6. Taniguchi, Y., *et al*., *labA*: a novel gene required for negative feedback regulation of the cyanobacterial circadian clock protein KaiC. *Genes Dev* **21**, 60-70 (2007).
7. Murayama, Y., Oyama, T., & Kondo, T. Regulation of circadian clock gene expression by phosphorylation states of KaiC in cyanobacteria. *J Bacteriol* **190**, 1691-1698 (2008).
8. Kutsuna, S., Nakahira, Y., Katayama, M., Ishiura, M., Kondo, T. Transcriptional regulation of the circadian clock operon *kaiBC* by upstream regions in cyanobacteria. *Mol Microbiol* **57**, 1474-1484 (2005).
