# Supplementary material for: Damped circadian oscillation in the absence of KaiA in Synechococcus
Source: Nat Commun. 2020 May 7;11:2242. doi: 10.1038/s41467-020-16087-x (PMC7205874; doi:10.1038/s41467-020-16087-x)
Supplement: Supplementary file 3 — Reporting Summary [file 41467_2020_16087_MOESM3_ESM.pdf]

nature research

Corresponding author(s): Hideo Iwasaki
Last updated by author(s): Mar 31, 2020

Reporting Summary

Nature Research wishes to improve the reproducibility of the work that we publish. This form provides structure for consistency and transparency in reporting. For further information on Nature Research policies, see Authors & Referees and the Editorial Policy Checklist .

Statistics

For all statistical analyses, confirm that the following items are present in the figure legend, table legend, main text, or Methods section.

n/a	Confirmed	
☐	☒	The exact sample size (*n*) for each experimental group/condition, given as a discrete number and unit of measurement
☐	☒	A statement on whether measurements were taken from distinct samples or whether the same sample was measured repeatedly
☒	☐	The statistical test(s) used AND whether they are one- or two-sided<br>*Only common tests should be described solely by name; describe more complex techniques in the Methods section.*
☐	☒	A description of all covariates tested
☒	☐	A description of any assumptions or corrections, such as tests of normality and adjustment for multiple comparisons
☐	☒	A full description of the statistical parameters including central tendency (e.g. means) or other basic estimates (e.g. regression coefficient) AND variation (e.g. standard deviation) or associated estimates of uncertainty (e.g. confidence intervals)
☒	☐	For null hypothesis testing, the test statistic (e.g. *F*, *t*, *r*) with confidence intervals, effect sizes, degrees of freedom and *P* value noted<br>*Give P values as exact values whenever suitable.*
☒	☐	For Bayesian analysis, information on the choice of priors and Markov chain Monte Carlo settings
☒	☐	For hierarchical and complex designs, identification of the appropriate level for tests and full reporting of outcomes
☒	☐	Estimates of effect sizes (e.g. Cohen's *d*, Pearson's *r*), indicating how they were calculated

Our web collection on statistics for biologists contains articles on many of the points above.

Software and code

Policy information about availability of computer code

Data collection	The codes of two mathematical models in Fig. 6 are available at https://github.com/hito1979/NatCommun2020.
Data analysis	For densitometric analysis of Western blot and Native PAGE, we used imageJ (ver. 1.52j).<br>For the optimization of the fitting in Figs. S3 and S4, we used lsqcurvefit subroutine of the MATLAB Statistical Toolbox (Mathworks, R2019a).

For manuscripts utilizing custom algorithms or software that are central to the research but not yet described in published literature, software must be made available to editors/reviewers. We strongly encourage code deposition in a community repository (e.g. GitHub). See the Nature Research guidelines for submitting code & software for further information.

Data

Policy information about availability of data

All manuscripts must include a data availability statement. This statement should provide the following information, where applicable:
- Accession codes, unique identifiers, or web links for publicly available datasets
- A list of figures that have associated raw data
- A description of any restrictions on data availability

The source data underlying Figs 1-5 and Supplementary Figs 1-9 are provided as a Source Data file.

Field-specific reporting

Please select the one below that is the best fit for your research. If you are not sure, read the appropriate sections before making your selection.

Life sciences study design

All studies must disclose on these points even when the disclosure is negative.

Sample size	No sample size calculation was performed. We chose as many sample sizes as those in previous studies such as Hosokawa et al., 2013 PNAS, https://doi.org/10.1073/pnas.1302243110.
Data exclusions	No data exclusions.
Replication	Bioluminescences of kaiA-less mutants were measured at least three times. Western blots were performed three times. Native PAGE experiments were performed two times. We comfirmed that all attempts at replication were successful. Only the additional data shown on Fig S1 are based on n=1, upon request from the reviewer.
Randomization	This study does not involve subjects that require randomization. We used each cloned cultural strain.
Blinding	This study does not involve subjects that require blinding. Instead, we performed appropriate control strains or samples for experiments.

Reporting for specific materials, systems and methods

We require information from authors about some types of materials, experimental systems and methods used in many studies. Here, indicate whether each material, system or method listed is relevant to your study. If you are not sure if a list item applies to your research, read the appropriate section before selecting a response.

Materials & experimental systems

n/a	Involved in the study	
☐	☒	Antibodies
☒	☐	Eukaryotic cell lines
☒	☐	Palaeontology
☒	☐	Animals and other organisms
☒	☐	Human research participants
☒	☐	Clinical data

Methods

n/a	Involved in the study	
☒	☐	ChIP-seq
☒	☐	Flow cytometry
☒	☐	MRI-based neuroimaging

Antibodies

Antibodies used	Peroxidase-Linked Secondary Antibodies (ECL^TM Anti-rabbit IgG Horseradish Peroxidase linked whole antibody (from donkey) NA934V lot#321815 (Amersham Biosciences, UK)), anti-KaiA and anti-KaiC antisera
Validation	ECL^TM Anti-rabbit IgG Horseradish Peroxidase linked whole antibody (from donkey) NA934 (https://www.gelifesciences.co.jp/catalog/0428.html). Anti-KaiA and anti-KaiC antisera were used and validated in Iwasaki et al., 1999 and Iwasaki et al., 2002.
